# Relationships of diverse apoptotic death process patterns to mitochondrial membrane potential (Δψ_m_) evaluated by three-parameter flow cytometric analysis

**DOI:** 10.1007/s10616-012-9455-0

**Published:** 2012-06-06

**Authors:** Yuhgi Suzuki, Hiroo Hasegawa, Tomohiro Tsuji, Kazuto Tsuruda, Daisuke Sasaki, Kaori Ishihara, Kazuhiro Nagai, Katsunori Yanagihara, Yasuaki Yamada, Shimeru Kamihira

**Affiliations:** 1 Department of Laboratory Medicine, Nagasaki University Graduate School of Biomedical Sciences, 1-7-1, Sakamoto, Nagasaki City, 852-8501 Japan; 2 Technology and Product Development, Division of Diagnostic Reagent Development, Sysmex Co., 4-4-4 Takatsukadai, Nishi-ku, Kobe City, 651-2271 Japan

**Keywords:** Apoptosis, Mitochondrial membrane potential, Annexin-V, Flow cytometry, Death signal pathway

## Abstract

Recently, it has been proposed that novel methodologies are needed to re-evaluate apoptotic cell death, as studies of apoptosis have shown it to be a complex process. Since mitochondria are key regulators in cell death pathways, we developed a simultaneous 3-parameter flow cytometric analysis that incorporates the change in mitochondrial membrane potential (Δψ_m_) in an Annexin-V [for phosphatidyl-serine (PS)] and propidium iodide (PI) assay system (3 parameters with 4 colours), and evaluated the apoptotic process using various haematological malignant cell lines and death triggers. The present method enabled visualization of cell composition during apoptosis and captured complicated molecular events. For example, apoptotic cells that lost Δψ_m_ did not always externalize PS, while some late apoptotic cells had polarized Δψ_m_. The findings of unchanged PS-externalization and aberrant cell death suggest that there is no relationship of PS externalization and apoptosis with an unknown apoptotic mechanism. Based on PS-externalization, sensitivity to staurosporine, and the combination of cell lines and triggers, the apoptotic process was classified into 2 types. Importantly, most of our findings could not be observed by PS–PI and Δψ_m_ assays when independently performed. Our method may be useful for examining mitochondrial-related apoptosis and death signalling pathways, as well as screening novel apoptosis-inducing cancer drugs.

## Introduction

Apoptosis accounts for most instances of patho-physiological cell death. Although originally defined by morphologic features (Kerr et al. 1972), apoptosis is carefully regulated by a variety of pro-apoptotic and anti-apoptotic signals, as well as cell growth signals (Hengartner 2000). Mitochondria are recognized to be at the crossroad of cell death and survival, as they are involved in the generation of adenosine tri-phosphate (ATP) and radical oxygen species (ROS), and release of cytochrome-*c* (Eguchi et al. 1997; Kroemer and Reed 2000). Under normal physiological conditions, energy released during oxidation reactions in the mitochondrial respiratory chain is stored as a negative electrochemical gradient across the mitochondrial membrane and the mitochondrial membrane potential (Δψ_m_) is referred to as being polarized. Collapse of Δψ_m_ during apoptosis has been reported in a number of studies, leading to the general notion that depolarization of mitochondria is one of the first events to occur during apoptosis and a prerequisite for cytochrome-*c* release (Bossy-Wetzel et al. 1998; Heiskanen et al. 1999). In addition, many studies have also investigated loss of Δψ_m_ using lipophilic cationic dyes such as CMXRos (chloromethyl-X-rosamine), TMRE (tetramethylrhodamine), JC-1, DiOC6(3), DilC1(5), and rhodamine 123 (Ly et al. 2003; Hakem et al. 1998).

To detect apoptosis, it is common to examine the externalization of phosphatidyl-serine (PS) on dying cells using Annexin-V in combination with propidium iodide (PI) (PS–PI assay) (Vermes et al. 1995). A combination of PS–PI and Δψ_m_ assays is one choice for evaluating apoptotic changes, though those are rarely performed in a simultaneous manner (Rasola and Geuna 2001). Herein, we established a 3-parameter flow cytometric assay consisting of Δψ_m_ status, and Annexin-V and PI staining. Although the basic theory and techniques behind this method have been available for many years, they have not been integrated into a practical 3-parameter method (PS, PI, and Δψ_m_) of analysis (Martinez et al. 2010; Eray et al. 2001), and the method has not been fully evaluated or elucidated.

Our aim in the present study was not to only simply detect apoptosis, but also to evaluate the qualities and patterns of apoptosis using a 3-parameter analysis method as compared with a PS–PI assay. This new methodology incorporating a portion of mitochondrial function is expected to be useful for determining apoptosis and related cell death.

## Materials and methods

### Cell preparations

We used 5 malignant haematological cell lines (KK1, ST1, LMY1, Jurkat, and MOLT4), 2 leukemic cell lines (K562 and THP1), and 2 B-cell lines (Ramos and SKW6.4). The ATL cell lines KK1, ST1, and LM-Y1 were established in our laboratory (Yamada et al. 1998), and have tumor necrosis factor (TNF)-related apoptosis-inducing ligand (TRAIL) death receptors (DRs) and CD95, and are semi-sensitive to TRAIL and the anti-Fas monoclonal antibody (Maeda et al. 1999; Hasegawa et al. 2005). Other cell lines were obtained from the American Type Culture Collection (Rockville, MD, USA). ST1, LM-Y1, and MOLT-4 cells carry wild-type p53, while the others carry mutated p53 (Kamihira et al. 2009). KK1 and LMY1 are dependent on exogenously added IL-2, and were maintained in RPMI1640 medium supplemented with 10 % fetal bovine serum (FBS) and 0.5 U/mL of IL-2 (kindly provided by Takeda Pharmaceutical Company, Osaka, Japan). The other cell lines were maintained in RPMI 1640 medium supplemented with 10 % FBS.

### Reagents

Staurosporine (STS) and betulinic acid (BEA) were purchased from Calbiochem (La Jolla, CA, USA). They were dissolved in DMSO and STS to make stock solutions of 100 μM and 5 mg/mL, respectively. Anti-Fas was purchased from MBL (Nagoya, Japan) and dissolved in RPMI1640 medium to make a stock solution of 1 μg/mL. TRAIL was purchased from BIOMOL Research Laboratories (Plymouth Meeting, PA, USA) and dissolved in RPMI1640 medium to make a stock solution of 20 μg/mL. Z-VAD-fmk was purchased from MBL.

### Treatments with death triggers

Jurkat cells were treated with STS (final concentration, 0.1 μM), anti-Fas (2.5 ng/mL) (Maeda et al. 1999), TRAIL (400 ng/mL) (Hasegawa et al. 2005), or BEA (50 μg/mL) (Ehrhardt et al. 2004; Fulda 2009). THP1, Ramos, and MOLT-4 cells were treated with 1 μM STS. ST1 and KK1 cells were treated with 2 μM STS and 100 ng/mL of anti-Fas. LMY1 cells were treated with 0.5 μM STS and 50 ng/mL of anti-Fas. K562 and SKW6.4 cells were treated with 2 μM STS. The concentration of each trigger was adjusted so that cell death after 24 h was uniform. All cell lines were treated with the caspase inhibitor Z-VAD-fmk (50 μM) before treatment with the death triggers. Apoptotic features were observed for 24 h after culture in the presence of a trigger or Z-VAD.

### Three-parameter assay

Our method consists of a Δψ_m_ assay based on the viable mitochondria transmembrane dye 1,1′,3,3,3′,3′-hexamethylindodicarbocyanine iodide [DilC1(5)] (Enzo Life Science, Philadelphia, USA), and Annexin-V and PI (Franklin Lakes, NJ, USA) assays. When the mitochondrial membrane potential (Δψ_m_) is intact, the dye becomes concentrated in the mitochondrial matrix and fluorescence intensity is determined using flow cytometry. Disruption of Δψ_m_ significantly compromises DilCl(5) accumulation, which is detected as a decrease in fluorescence intensity (Lee et al. 1999). DilC1(5) was added at a final concentration of 5 nM to the culture system 20 min before harvest. Then, after being washed with PBS, the cells were stained with Annexin-V and PI, according to the manufacturer’s instructions. Flow cytometric identification of early and late apoptotic cells was performed according to the Annexin-V/PI method (Martinez et al. 2010; Vermes et al. 1995). We also used a caspase-3 assay kit (NucView 488; Biotium, Hayward, CA, USA), and kits for detecting the activities of caspase-8 and -9 (FLICA Apoptosis Detection kits; Immunochemistry Technologies LLC, Bloomington, MN, USA). Cell staining was evaluated using FACS Canto and FACS Diva software (BD Bioscience Immunocytometry Systems, San Jose, CA, USA).

## Results

### Concept of 3-parameter flow cytometric methodology

**Fig. 1:**
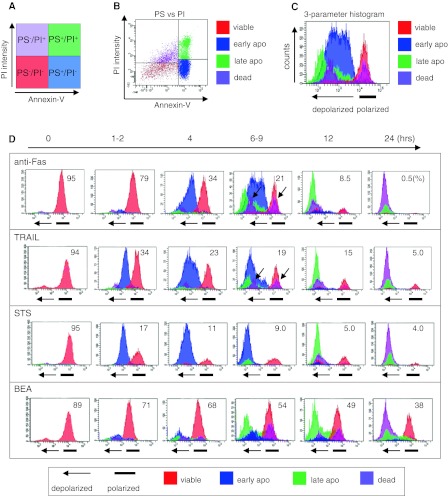
Three-parameter flow cytometer analysis incorporating changes in Δψ_m_ into Annexin-V [phosphatidyl serine (PS)] and propidium iodide (PI) cytograms. **a**, **b** After gating of cells stained in PI (*Y*-axis) and PS (*X*-axis) assays, each cell population was* coloured* *red* (*lower left* *quadrant*), *blue* (*lower right quadrant*), *green* (*upper right quadrant*), or *violet* (*upper left*), which corresponded to viable, early apoptotic, late apoptotic, and dead/unknown cells, respectively. **c** Three-parameter histogram. Most of the dying cell populations were seen in depolarized areas. **d** Sequential observations using a 3-parameter histogram of Jurkat cells treated with anti-Fas, TRAIL, staurosporine (STS), and betulinic acid (BEA). The initial depolarization time, peak patterns of each type of cell, and association with loss of Δψ_m_ varied with each trigger. The percentages of *red*-*coloured* cells (viable cells) are indicated in each panel. *Arrows* show *violet*-*coloured* cells

To conduct 3-parameter flow cytometric analysis, we used anti-Fas-treated Jurkat cells, which are known to show typical apoptotic changes (Scaffidi et al. 1998). After being treated with anti-Fas for 6 h, cells were stained with Annexin-V, PI, and DilC1(5). In a typical PS–PI assay, cells are subdivided into 4 main groups (viable, early apoptotic, late apoptotic, and dead) and PS-positive cells (or PS^+^ and PI^−^ cells) are considered to be apoptotic (Martinez et al. 2010). In the present study, 4 colours were determined using the results of the PS–PI assay (Fig. 1a). Cell populations that appeared in the PS^−^/PI^−^ (corresponding to viable cells), PS^+^/PI^−^ (early apoptotic cells), PS^+^/PI^+^ (late apoptotic or necrotic cells), and PS^−^/PI^+^ (dead or unknown cells) areas were coloured red, blue, green, and violet, respectively (Fig. 1a, b). Then, the cell populations were developed into a Δψ_m_ histogram based on DilC1(5) staining (Fig. 1c). This 3-parameter histogram successfully separated the cells into 2 fractions; those in polarized and depolarized areas, with the 4 colours maintained. Cells in polarized areas consisted of viable (red) cells. Collapse of Δψ_m_ resulted in depolarized Δψ_m_ and depolarized area cells that were early (blue) or late (green) apoptotic cells (Fig. 1c).

### A 3-parameter histogram of Jurkat cells treated with various death triggers

Jurkat cells treated with various death triggers under conditions that caused cell death after 24 h became uniform (Fig. 1d), then the apoptotic process was observed using a 3-parameter histogram. Most early apoptotic (blue) cell populations defined by the PS–PI assay were primarily distributed in depolarized areas within 6–9 h and showed 2 characteristic features. First, the time taken for the initial depolarization was shortest for STS, followed in order by TRAIL and anti-Fas, though the conversion from the early (blue) to late (green) apoptotic peak was nearly simultaneous. Similar results have been observed in previous studies, though the PS–PI and Δψ_m_ assays were performed separately (Vander Heiden et al. 1997; Waibel et al. 2007; Koya et al. 2000). Second, the width of the early apoptotic (blue) peak in a depolarized area became wider in the same time order (Fig. 1d). These results indicate that the depolarization caused by each trigger was heterogeneous and suggest that the death process in Jurkat cells differs with the type of trigger. BEA was less effective than the other triggers and did not cause cell death. Jurkat cells treated with BEA progressed from the early (blue) to late (green) apoptotic phase before complete loss of Δψ_m_. As a result, viable (red) and late apoptotic (green) cells co-existed for a long period of time. Interestingly, dead (or unknown) cells (violet) had 2 populations that were distributed into polarized and depolarized areas (arrows in Fig. 1d). As shown in Fig. 1b, PS^−^ cells after apoptosis and viable cells were mixed in this dead cells (violet) area. Thus, it is reasonable that these populations were distributed into polarized and depolarized areas.

### Time course analysis of various apoptotic processes in relation to loss of Δψ_m_

**Fig. 2:**
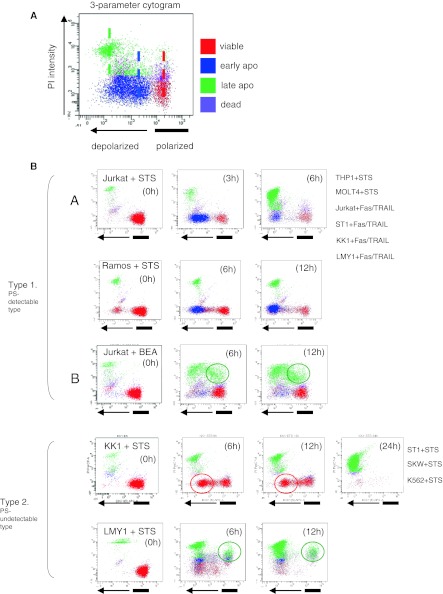
Three-parameter cytograms. Cell populations defined by the PS–PI assay were incorporated into Δψ_m_ (*X*-axis) and PI (*Y*-axis) cytograms. **a** Representative 3-parameter cytogram. *Coloured bars* indicate the peak fluorescence intensity of each cell composition. **b** Cell lines were treated with death triggers as indicated. Time course analyses of polarized (*upper column*) and depolarized (*lower column*) cells using a 3-parameter cytogram. The patterns were classified into 2 major types; PS-externalization-detectable (Type 1) and undetectable (Type 2). Type 1 was further subclassified into 2 subtypes; subtype A, typical apoptotic changes, and subtype B, complicated type with or without mitochondrial function. Type 2 cells showed no emergence of *blue*-*colored* cells (early-apoptotic) despite depolarization, though apoptotic cell death occurred, as shown by the *green cells* (late-apoptotic)

Figure 2a presents a 3-parameter cytogram with Δψ_m_ as the *x*-axis and PI as the *y*-axis, incorporating the 4 colours from the PS–PI assay. We focused on the cell compositions in polarized and depolarized areas, and observed the time course of changes in colour for each area. For example, in STS-treated Jurkat cells, viable cells (red) in polarized areas disappeared after 6 h. Similarly, early apoptotic cells (blue) in depolarized areas changed to late apoptotic cells (green). We found this time-course analysis of 3-parameter cytogram findings useful to understand the cell death pattern in relation to loss of Δψ_m_.

The diverse apoptotic patterns could be classified into 2 major types when we focused on STS-treated cells (Fig. 2b), with the main difference between Types 1 and 2 the presence or absence of PS-externalization. Typical apoptotic changes shown in Fig. 1d (anti-Fas, TRAIL, or STS) correspond to Type 1A. Type 1B was observed only in BEA-treated Jurkat cells, and Type 2 cells did not show PS-externalization and remained viable (red). Cells in a depolarized area tended to directly change from viable (red) to late apoptotic (green). Of note, the features of each type were not dependent on cell type, but rather were flexible. ST1, KK1, and LYM1 cells were Type 1 when triggered by DRs, then became Type 2 with STS. Although these cells differed in sensitivity to DRs and STS (poor sensitivity with STS), an unexpected finding was that viable (red) cells appeared in depolarized areas of STS-treated cells (red circles in Fig. 2b). We also observed that late apoptotic (green) cells appeared in polarized areas among BEA-treated Jurkat cells or STS-treated LM-Y1 cells (green circles in Fig. 2b).

### Z-VAD inhibition of various apoptotic processes

**Fig. 3:**
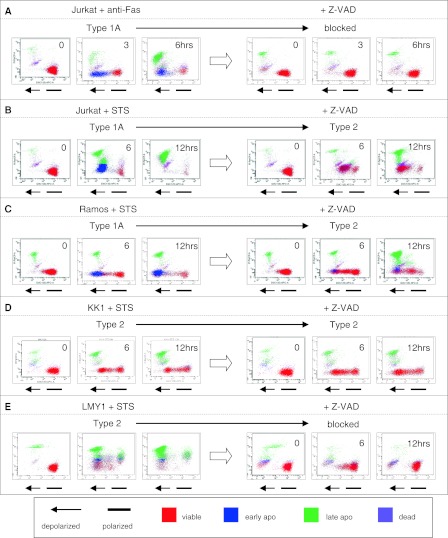
Comparison of cell death patterns with or without Z-VAD. Time course analysis and representative data from 3-parameter cytograms are shown. Most Type 1 cells lines changed into Type 2, namely *blue-colored* cells (early-apoptotic) disappeared and underwent apoptosis. **a** Depolarization is blocked by Z-VAD. **b, c** The *blue*-*colored* cell population (early-apoptotic) disappeared after treatment with Z-VAD and underwent apoptosis. **d** Most Type 2 cell lines showed no change. **e** Depolarization was blocked by Z-VAD in STS-treated LMY1 cells

Next, to examine the mechanistic backgrounds of the various apoptotic processes, Z-VAD inhibition tests were performed using Jurkat, Ramos, KK1, SKW, and LMY1 cells, and observed using 3-parameter cytogram findings. Jurkat cells were Type 1 when treated with anti-Fas and STS, whereas the addition of Z-VAD resulted in different cell death patterns (Fig. 3a, b). The apoptotic change was nearly blocked in anti-Fas-treated cells, but not in STS-treated cells, which changed to Type 2 with Z-VAD. Similarly, Ramos changed from Type 1 to Type 2 (Fig. 3c), KK1 and SKW6.4 cells remained unchanged (Fig. 3d), and apoptosis was nearly blocked by Z-VAD in LYM1 cells (Fig. 3e). Since Z-VAD is a cell-permeant pan-caspase inhibitor, we speculated that there are caspase-dependent (Fig. 3a, e), partly-dependent (Fig. 3b, c), and caspase-independent (Fig. 3d) cell-death pathways.

### Options for 3-parameter flow cytometric analysis: caspase activity assays

**Fig. 4:**
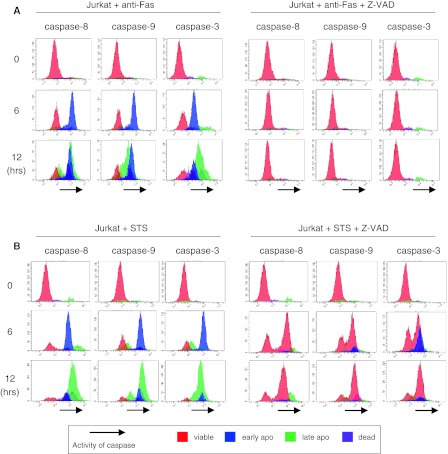
Optional 3-parameter flow cytometric analysis based on caspase activities. **a** Activities of caspases in anti-Fas-treated Jurkat cells with or without inhibition by Z-VAD. After cells were stained for a PS–PI assay,* coloured* cells were included in a 3-parameter histogram with the activities of caspases as the *X*-axis. **b** Activities of caspases in STS-treated Jurkat cells with or without inhibition by Z-VAD

We also constructed assays for caspase activities based on our 3-parameter flow cytometric method. Instead of Δψ_m_, the activities of caspases-8, -9, or -3 were analyzed using simultaneous assays of PS–PI. Those of caspases in anti-Fas-treated Jurkat cells increased within 3 h, and the activated cells appeared early apoptotic (blue) and then turned late apoptotic (green) (Fig. 4a, left). Activities of caspases in these cells were completely blocked by Z-VAD and the peaks of viable (red) cells showed no change (Fig. 4a, right). Similarly, all caspases were activated in STS-treated cells within 3 h (Fig. 4b, left). Importantly, STS-treated cells and viable (red) cells showed caspase activities (Fig. 4b, right).

### Three-parameter assays enable invisible apoptotic cells to be captured

**Fig. 5:**
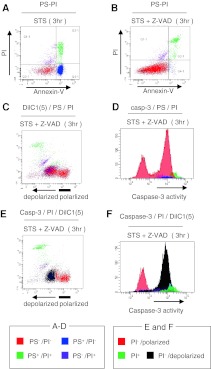
Combined use of assays in 3-parameter flow cytometric analysis. **a** PS–PI assay of STS-treated Jurkat cells. **b**–**f** STS-treated Jurkat cells with inhibition by Z-VAD. **b** PS–PI assay. **c** Three-parameter Δψ_m_ analysis (Dilc1(5)/PS/PI). **d** Three-parameter caspase-3 analysis (caspase-3/PS/PI). **e** Another 3-parameter Δψ_m_ analysis (caspase-3/PI/Dilc1(5), *X*-axis is Δψ_m_), in which depolarized cells are shown as *black*. **f** The *X*-axis in **e** is changed from Δψ_m_ to caspase-3

Next, we focused on inhibition of caspases by Z-VAD in STS-treated Jurkat cells. The results of the PS–PI assay indicated that Z-VAD attenuated apoptotic changes, remaining as a viable (red) cell population in PS^−^/PI^−^ areas (Fig. 5a, b). In contrast, 3-parameter assays (Dilc1(5)/PS/PI, or caspase-3/PS/PI) revealed that viable (red) cells consisted of 2 populations with different features of Δψ_m_ and caspase-3 activities (Fig. 5c, d). Furthermore, when we used a different 3-parameter assay [caspase-3/PI/Dilc1(5)] and coloured the depolarized cells black (Fig. 5e), it was clear that depolarized cells had active caspase-3 (Fig. 5f). These results confirmed that most of the viable (red) cells seen in Fig. 5b were apoptotic and our assay captured hidden apoptotic cells. Meanwhile, increasing concentrations of Z-VAD did not inhibit depolarization of Δψ_m_ or caspase activities in these cells (data not shown). These results suggest that neither the depolarization of Δψ_m_ not inhibited by Z-VAD nor activation of caspases in STS-treated Jurkat cells was due to a lack of Z-VAD.

## Discussion

Although numerous studies have investigated apoptosis, the expansion of apoptotic research has made it difficult to understand common concepts, including cell death patterns. Therefore, it is necessary to re-evaluate cell death patterns and present novel methodology from a new conceptual viewpoint (Kroemer et al. 2009). In fact, a functional classification of regulated cell death modes is proposed including necrotic cell death as well as apoptosis (Galluzzi et al. 2012). Recently introduced methods for evaluating apoptosis include fluorescence cross-correlation spectroscopy (Kim et al. 2007), electrochemical-based biosensors (Liu et al. 2009), and micro-fluidic devices (Valero et al. 2005), each with advantages and disadvantages (Martinez et al. 2010). Although conventional methods also have some disadvantages, PS–PI and/or Δψ_m_ assays are predominantly used in analyses of apoptosis, likely because they provide concise results. In the present study, we developed a simple methodology by combining conventional methods for apoptotic estimation based on mitochondrial functions. We conducted 3-parameter flow cytometric analyses with 4 colours and evaluated the apoptotic process from various aspects.

First, we observed a trigger-specific death process in Jurkat cells using 3-parameter histogram findings and found 2 major features; a difference in the timing of initial depolarization and heterogeneous distribution of early apoptotic (blue) cells in depolarized areas (wide or sharp peak). These results indicate that substantially different events occurred in response to each trigger in the same cell line. Thus, 3-parameter histogram findings may be suitable for examining the mechanism of apoptosis.

We also observed the time-course changes of Δψ_m_ using 3-parameter cytogram findings. Despite diversity or differences in response patterns between cell type and type of death trigger, diverse death patterns shown by the 3-parameter cytogram were classified into Types 1 and 2. Interestingly, the features of Types 1 and 2 were not dependent on cell type, but rather were flexible. ST1, KK1, and LYM1 cells were Type 1 when triggered by DRs, and then changed into Type 2 when using STS. Another characteristic of the 3-parameter cytogram findings was detection of aberrant cell populations based on localization site and/or colour. We found late apoptotic (green) cells in polarized areas among BEA-treated Jurkat cells and STS-treated LM-Y1 cells. These cells likely employ an unknown death signalling pathway different than mitochondrial membrane depolarization and such an aberrant cell death is not verified yet (Galluzzi et al. 2012). BEA has been reported to have complicated death signalling, resulting in unusual death, as described by Fulda (2009).

Another unexpected finding in the present study was the appearance of viable (red) cells among STS-treated cells in depolarized areas. It is known that exposure to PS does not occur in early apoptotic stages and is scarcely detectable in some types of cells by Annexin-V (Gatti et al. 1998). Furthermore, previous reports have pointed out that depolarized cells are not always linked to apoptotic cells. Kim et al. (2006) reported transient or reversible depolarized cells. However, aberrant viable (red) cells changed directly to late apoptotic (green) cells and caused cell death in our study. This phenomenon has not been clearly elucidated or fully investigated. Such cells may undergo accelerated apoptosis via a loss of Δψ_m_ rather than PS externalization. These events are scarcely detectable by a PS–PI assay and cell composition (colour) is not revealed by a common Δψ_m_ assay. In this regard, the individual weaknesses of the PS–PI and Δψ_m_ assays seem to be covered by the strength of the other one in our 3-parameter analysis. Interestingly, novel cell death modes such as parthanatos and netosis, which showed irreversible Δψ_m_ dissipation but not PS exposure, were reported and were considered as a kind of regulated necrosis and/or autophagic cell death (Galluzzi et al. 2012; Wang et al. 2009; Mihalache et al. 2011).

We tentatively concluded that caspase-dependent and partially dependent, as well as caspase-independent cell-death pathways are shown in Z-VAD tests. However, there is a number of unanswered questions regarding caspase-dependent and -independent cell death (Chipuk and Green 2005; Belmokhtar et al. 2001; Nicolier et al. 2009). Several reports have indicated the existence of a caspase-independent apoptotic pathway involving STS-induced cell death. In addition, the actual mechanism of PS-externalization remains to be elucidated (Balasubramanian et al. 2007; Smrz et al. 2007). It has also been noted that PS exposure is not always specific in apoptosis, and additional precise biochemical, enzymatic, and morphological events should be assessed (Kroemer et al. 2009; Martinez et al. 2010). In the present study, Z-VAD never failed to inhibit PS exposure regardless of the conditions, while it failed to inhibit the execution of apoptosis in a variety of conditions. It is possible that Z-VAD blocks the translocation of PS, but cannot inhibit apoptotic death itself, especially in STS-treated cells, which suggests that STS has a death pathway(s) unrelated to Z-VAD functions. These features may correspond to ‘Caspase-independent intrinsic apoptosis’ described in a recent review (Galluzzi et al. 2012). This also raises the question of whether a condition not inhibited by Z-VAD is caspase independent.

We conducted another 3-parameter analysis using a caspase activity assay instead of Δψ_m_. During typical apoptotic changes, such as those seen in anti-Fas-stimulated Jurkat cells, the results of our 3-parameter caspase activity assays corresponded completely to our 3-parameter histogram findings. Anti-Fas can induce apoptotic death of CD95-positive cells, such as Jurkat, KK1, ST1 and LMY1 cells, which was completely inhibited from depolarization or activation of caspases in each cell type by Z-VAD, leading to cell survival. This shows that death signalling can be inhibited by Z-VAD and/or is caspase dependent. Importantly, in PS–PI assays of STS-treated Jurkat cells, Z-VAD attenuated apoptotic changes, while we also noted that viable (red) cells were distributed in depolarized areas and those cells harboured active caspase-3. These results are not consistent with previous reports that suggested that early loss of Δψ_m_ occurs independently of caspase activation (Ly et al. 2003; Marzo et al. 2001; Mahyar-Roemer et al. 2001). Kroemer et al. (2009) pointed out that the term ‘Z-VAD inhibitable’ should be preferred to ‘caspase-dependent’ and that it should be accepted that caspase-independent mechanisms can cooperate with (or substitute for) caspases in the execution of lethal signalling pathways. In this regard, our results indicate that a condition not inhibited by Z-VAD is not synonymous with a caspase-independent condition. Recently, Galluzzi et al. (2012) pointed out that chemical inhibition of caspases rarely confers long-term cytoprotective effects and only delays the execution of cell death, which eventually can even exhibit morphological features of necrosis. In this regard, our methods may be good at capturing incomplete inhibition of apoptosis. Although a flow cytometric assay system for caspase activity has been available for many years, results of that performed simultaneously with a PS–PI or Δψ_m_ assay have been rarely reported (Fox and Aubert 2008). In the present study, we clearly observed caspase activity in each of the 4 different coloured cell populations, while such results cannot be observed using 2-colour flow cytometric analysis or western blotting of whole cell lysates. Our findings suggest that a combination of caspase activity assays is a good option for 3-parameter assays.

Apoptosis is a representative cell death pattern and aberrant apoptosis is causatively associated with diseases. Most chemotherapeutic drugs have been shown to activate common apoptotic pathways in target cells. Since our methodology enables visualization of cell composition during the process of apoptosis, it captures and screens complicated molecular events. In this study, we observed some previously unnoticed cell populations, though some findings are left unexplained or raise further questions. It is interesting that the PS–PI and Δψ_m_ assays seem to cover for the disadvantages of the other one in 3-parameter assays, which suggests that this is a novel methodology comprised of conventional methods. In future studies, 3-parameter assays may be useful for screening mitochondrial-related apoptosis and various regulated cell death patterns, as well as novel apoptosis-inducing anti-cancer drugs.
